# The Silver Agri Age project in Italy: a Montessori-inspired social intervention with older adults with mild cognitive impairment. Single-group pre-post pilot study protocol

**DOI:** 10.3389/fpubh.2025.1561263

**Published:** 2025-05-16

**Authors:** Elena Gambella, Lucio Lombardi, Diletta Cicconi, Alessandra Raccichini, Lucia Paciaroni, Cinzia Giuli, Anna Rita Bonfigli, Paolo Fabbietti, Giuseppe Pelliccioni, Cristina Gagliardi

**Affiliations:** ^1^Centre for Socio-Economic Research on Ageing, National Institute of Science and Health for Ageing (IRCCS INRCA), Ancona, Italy; ^2^International Montessori Centre, Perugia, Italy; ^3^Department of Neurology, National Institute of Science and Health for Aging (INRCA-IRCCS), Ancona, Italy; ^4^Department of Neurology, National Institute of Science and Health for Aging (INRCA-IRCCS), Fermo, Italy; ^5^Centre for Biostatistics and Applied Geriatric Clinical Epidemiology, Italian National Research Center on Aging (IRCCS INRCA), Cosenza, Italy; ^6^Centre for Socio-Economic Research on Ageing, Italian National Research Center on Aging (INRCA-IRCCS), Ancona, Italy

**Keywords:** nature, Montessori, psychosocial intervention, social farming, mild cognitive impairment

## Abstract

**Introduction:**

According to literature, an estimated percentage of 10–15% of people diagnosed with Mild Cognitive Impairment each year develop Alzheimer’s dementia. Prevention and non-pharmacological treatments play an important role in dealing with this emergency. In this regard, literature has highlighted how exposure to nature, participation in horticultural and cognitive activities, and adopting a Montessori approach are useful to counteract cognitive decline and promote well-being. Therefore, the Silver Agri Age pilot study will test a Montessori-inspired social intervention that will be carried out on farms of the Marche Region (Central Italy) authorized for social agriculture and aimed at an older adult population with mild cognitive impairment in order to improve their well-being and quality of life. This paper describes the pilot study protocol and main outcome.

**Methods:**

Four older adult people will be involved for each of the three farms participating in the study, for a total of 12 participants. The inclusion criteria will be age ≥ 55, Mini-Mental State Examination ≥ 24, ability and willingness to sign informed consent. The evaluation will focus on the assessment of the person’s emotional well-being, life quality, and cognitive status. To evaluate the feasibility of the pilot project, the quality of participants’ engagement in the activities and the satisfaction of the subjects and their caregivers with the project will also be assessed.

**Discussion and conclusion:**

During each three-month phase of the project different social farming activities will be proposed. Activities will also include sensory and cognitive stimulation and socialization within the farm. Since according to literature, participation in Montessori-based programs and exposure to nature generate positive effects, the emotional well-being of participants will be assessed as a primary outcome and life quality as a secondary outcome. Additionally, we believe that promoting participation in progressively challenging activities and autonomy may help stabilize cognitive decline. Therefore, cognitive level will also be assessed as a secondary outcome. Ultimately, the pilot study will provide insights into the possibility of integrating a prototype non-pharmacological intervention aimed at improving the well-being and quality of life of people with MCI into the dementia prevention service system.

**Clinical trial:**

ClinicalTrials.gov Identifier: NCT06754202.

## Introduction

Rising life expectancy is contributing to a rapid increase in the number of older people worldwide and associated with a higher prevalence of chronic diseases such as dementia (1). According to the literature, dementia is one of the greatest global public health and social care challenges of our time and currently has no known pharmacological cure (1). This highlights the crucial role of prevention and non-pharmacological treatments as a strategy to address the emergency (1). American studies show that mild cognitive impairment (MCI) represents a transitional stage between healthy aging and dementia, affecting 10–15% of the American population over the age of 65 (2) and 19.7% of the global population over the age of 50 (3). In Italy, prevalence estimates vary from 3.2 to 24.5% in different studies (4). MCI is characterized by a slight impaired in one or more cognitive domains (e.g., memory, executive function, attention, language, and visuospatial skills), which does not interfere with psychosocial functioning and not meet the diagnostic criteria for dementia (5). According to the literature over 46% of individuals with MCI around the world progress to clinical dementia within 3 years (3). Moreover, studies on the American population indicate that approximately 8–15% of individuals with MCI progress to dementia every year, up to 80% develop dementia within 6 years (5). However, an Italian study found that 74% of individuals with MCI remained stable 1 year after diagnosis, and 10% reverted to normal cognitive functions (6). As a result, the National Dementia Observatory of the Italian National Institute of Health (ISS) has underlined the importance of promptly addressing this condition in order to delay its progression and conversion to dementia, thereby also reducing its social and economic impact (7). According to the literature it is possible to reduce – or at least delay – the risk of conversion from MCI to dementia by targeting a range of lifestyle-related risk factors, including levels of physical activity, social engagement, diet, mental well-being and cognitive stimulation (8–10). Recent MCI guidelines recommend group psychosocial interventions that support the adoption of healthier lifestyles to promote daily functioning and well-being in people with MCI (2). The guidelines also stress the importance of learning coping strategies that help people with MCI to relax and manage the challenges they face (2). In this context, the literature points out that spending time in natural environments offers individuals the opportunity to reflect on unresolved issues and to relax (11). These two responses – reflection and relaxation – may be considered valuable coping strategies for addressing the challenges associated with cognitive decline (2, 11).

Exposure to nature is associated with increased happiness and seems to be useful to counteract psychological and social problems resulting from cognitive decline such as social withdrawal, loneliness, disengagement (11, 12). Indeed, a recent study shows involvement in social farming promotes positive social relationships and improve engagement in daily and recreational activities in healthy older people (13).

Moreover, a recent Systematic Review shows that horticultural therapy and farming activities can be a feasible way to reduce the depressive symptoms, agitation and provide a sense of self-worth and social connection for people with dementia (14). This aspect is very important given that according to the literature increasing positive affect and decreasing negative affect in older people seem to be associated with a reduced risk of mortality (15). A review of several studies shows that connecting with nature is associated with an improved subjective well-being, including an improved emotional functioning and satisfaction with life (16–18). Subjective well-being appears to be linked with good health and better social relationships which are important for healthy aging (18, 19). Moreover, the environment appears important not only for the benefits of contact with nature but also in terms of specific properties of the setting that support various types of activities. In fact, according to the literature interacting with an environment that supports physical, cognitive, and social activities increases cognitive reserve and attenuates the effect of neuropathology, improving the person’s quality of life (10, 20, 21). For this reason, it is important to create an environment that can support independence in carrying out daily activities by eliminating obstacles and making it more suitable for the residual abilities of the person with cognitive decline (22).

To preserve social and cognitive engagement several studies underline how useful it is to adapt the Montessori approach to the needs of older adult people and in particular to those with dementia (23). According to the literature, activities based on Montessori principles generate positive affect in older people, healthy eating behaviors, prosocial behavior, and constructive engagement, as well as support cognition (24). Several studies highlight in the flexibility of the Montessori approach as a feature that allows it to adapt well to the treatment people with dementia in the different phases of the disease, improving the quality of life of both people with dementia and those who take care of them (25).

Considering everything highlighted, the Silver Agri Age project aims to structure a Montessori-inspired social intervention, to be implemented on registers social farms and targeted at older adults with mild cognitive impairment. This project therefore aims to improve the subjective well-being of people with MCI by helping them enhance their emotional well-being and quality of life, feel less alone and become more capable of coping with problematic situations. Finally, in our project we would like to set up an innovative pilot experience that extends into new contexts – i.e., the agricultural one – and to new population groups – i.e., subjects with MCI – by adapting programs already tested in countless studies on healthy older adult people or in non-agricultural contexts.

## Methods and analysis

### Study design

**Figure 1:**
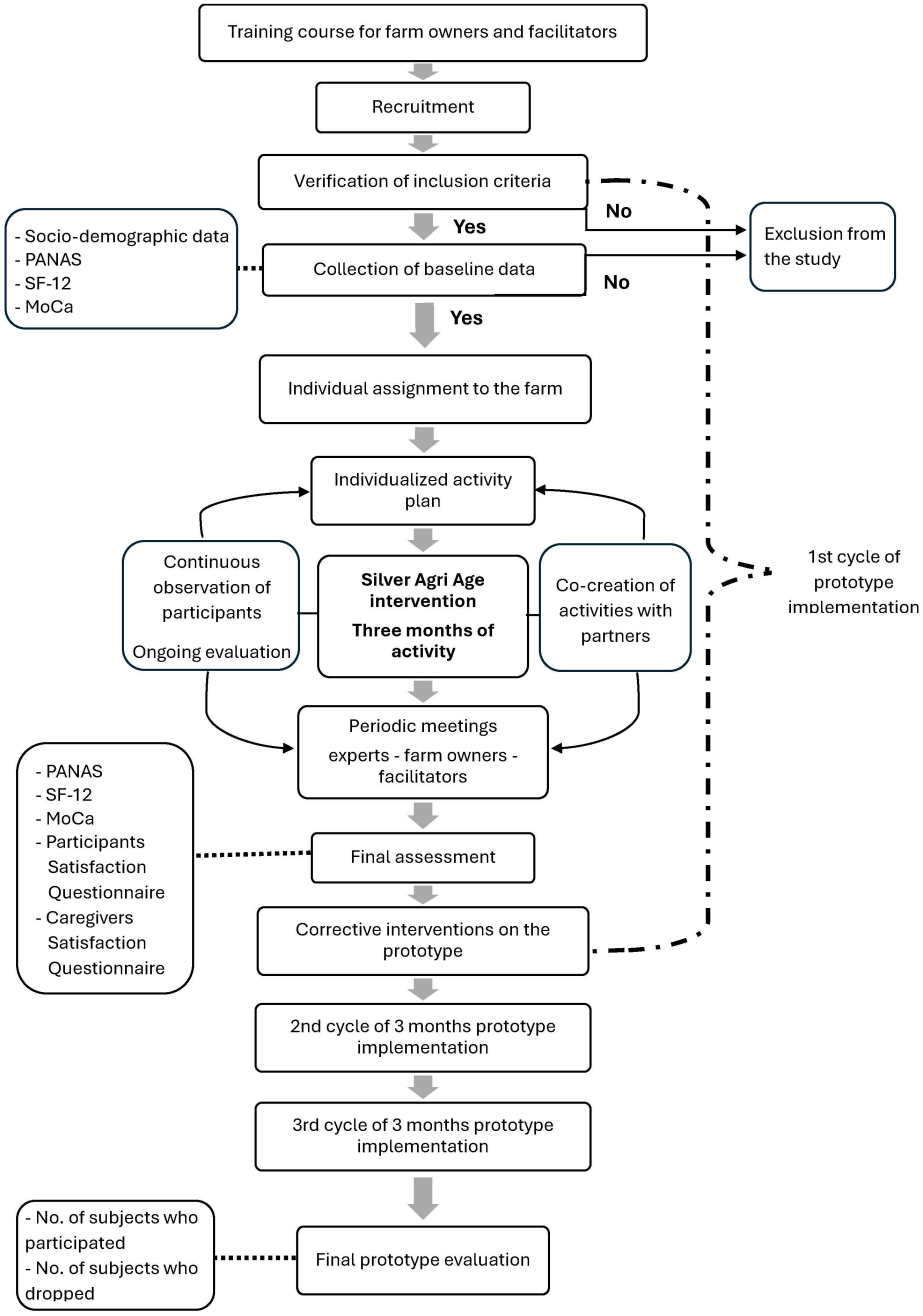
Project flowchart.

A pre-post pilot study will be conducted to test a single-arm social intervention with older adult subjects with mild cognitive impairment. Participants will undergo a questionnaire evaluation at baseline and at the end of each 3-month program. The study process is outlined in the flowchart shown in Figure 1.

### Study population

For the purposes of this pilot study, a limited number of participants will be selected to evaluate this intervention for this type of subject and to assess the feasibility of the intervention and participant satisfaction. The sample therefore consists of 4 participants for each of the three farms involved in the study, for a total of 12 participants overall. According to the literature, this is the maximum number of individuals with MCI that can be accommodated on each farm under the exclusive supervision of non-healthcare facilitators. Eligible participants are male or female and at least 55 years of age at the time of baseline assessment. These subjects can only be included in the study if they have a diagnosis of MCI (26, 27).

Exclusion criteria are applicable to participants who have a more severe level of cognitive impairment and physical conditions that prevent participation in farm activities (e.g., severe visual impairment and motor deficits). The active participation in another research study is also an exclusion criterion.

All eligibility criteria at the time of baseline assessment are summarized in Table 1.

**Table 1:** Eligibility criteria.

Inclusion criteria
55 years of age or older;
male and female;
diagnosis of mild cognitive impairment (MCI);
Mini Mental State Examination ≥ 24;
ability and willingness to sign informed consent.
Exclusion criteria
Mini Mental State Examination <24;
severe visual impairment;
inability to walk without help or assistance, Tinetti’s scale <20;
refusal to provide informed consent;
participation in another intervention study.

### Study settings

Participants will be recruited from both clinical and non-clinical environments.

Participants from clinical settings will be recruited through the outpatient clinics of the INRCA Hospitals in Ancona and Fermo, as well as the Territorial Health Authorities of Ascoli and Fermo. Participants from non-clinical settings will be recruited through local parishes, voluntary associations, and pharmacies that will help promote the project activities.

This study will take place at three agricultural companies registered in the EROAS list, List of Social Agriculture Operators of the Marche Region (Italy). Registration on this list guarantees compliance with the requirements established by the regional resolution on social agriculture (DGR 345/2016) and regional law no. 21/2011 (Regional provisions regarding the multifunctionality of the agricultural company and diversification in agriculture).

### Project components

#### The social agriculture or social farming

Although few internationally interventions currently exploit nature as a strategy to promote mental health, there is already evidence to support the effectiveness of social agriculture programs for older adults (16). According to the literature social farming or social agriculture uses the resources offered by farms (e.g., animals, plants …) to provide social care services and promote well-being as well as mental and physical health (28, 29). The literature also suggests that exposure to nature or green space improves physical and mental health in older people (30). In this regard, according to the Attention Restoration Theory (ART), interaction with natural environments produce greater benefits than interaction with urbanized ones, as it requires less effort to restore attention skills (11, 31). Indeed, nature appears to be particularly useful because it allows individuals to escape from daily stresses, experience expansive spaces and contexts and engage in activities in line with each person’s intrinsic motivations (11). Given the attention deficits often associated with MCI, this nature-related benefit is particularly relevant (2). Finally, engaging in nature-based activities, characterized by slow rhythms and daily routines, helps individuals rediscover a sense of security that promotes well-being (32). Multiple studies have emphasized that social farming is a highly complex intervention comprising multiple activities and involving many client groups with differing needs (29). The main difference between social farming and regular care services for people with MCI lies in the agricultural, non-medicalised setting where the activities are carried out (28). This difference helps reduce stigma and encourages the involvement of individuals in the proposed activities (28). In particular, the added value for the project can be summarized in the following five key points:

- Physical activity. Because most activities on the farm and in nature require more physical effort, farms offer older people with cognitive impairments more opportunities to be physically active than time spent in indoor settings (29);
- Sensory stimulation using the environment/nature and farm activities, including animal care and the sensory qualities of typical farm products such as texture and flavor (33);
- Orientation to reality (ROT). Engaging in activities and participating in seasonal events supports orientation in time and space by linking experiences to natural temporal cues (33, 34);
- Maintenance of social interaction and relationship-building, fostered through collaborative activities in the natural environment of the farm (16, 28, 29);
- Reminiscence: Environmental stimuli serve as powerful triggers for memories of habits and emotions tied to personal history and rural traditions, which become topics of discussion in group settings (33).

In the project, social agriculture will include different agricultural activities (horticulture or livestock breeding and care) and other activities such as gardening, herb conservation, woodwork, using land products to prepare meals, with different levels of support provided according to needs of each user.

#### Montessori approach

With reference to Montessori educational philosophy, the principles that will be used to shape the project are:

- identifying an interesting and appropriately challenging activity for the individual based on his or her skills;
- making use of familiar materials from everyday environments;
- breaking the activity down into small steps to enable people to complete it successfully and independently;
- inviting people to complete the task themselves and providing cues for the person to self-correct
- giving the person the possibility of “free choice.”

Giving the person the possibility to choose whether to participate or not in the proposed activities is very important because it recognizes the right to self-determination that is too often taken away from them in everyday life (35). The possibility of “free choice” improves the sense of dignity, increases self-esteem and reduces the likelihood that participants will refuse to participate in the program (35, 36).

The usefulness of applying these principles to the non-pharmacological treatment of older adult people with dementia residing in nursing homes for the older adult with dementia has already been discussed and demonstrated by J. Camp. Indeed, the Montessori-based dementia program is a non-pharmacological intervention based on a person-centered approach which has proven useful in supporting the autonomy of people with dementia (23, 24, 37).

Moreover, according to literature, participating in challenging and meaningful activities and successfully completing them induces a sense of satisfaction and appreciation for the experience, regardless of the level of the cognitive impairment (38). Therefore, since individuals with MCI experience less cognitive deterioration than those with dementia, the Silver Agri Age project aims to draw from this program the salient principles to apply them to subjects with MCI in organizing the project activities.

Therefore, project activities will be customized to fit the cognitive capacity and specific characteristics of the individual and to encourage active engagement and direct contact with the social and physical environment. The Montessori principle of “free choice” will be the one that will most inspire the entire project. In particular the person first will have the possibility to choose whether to join the project or not and then, throughout the course of the project, the person will always have the possibility to choose the level of commitment and the way of participation. The proposal of activities by the facilitators will be inviting and not coercive and the activities can also be proposed directly by the subjects involved. Furthermore, the activities will be designed as a stimulus to maintain and strengthen daily life skills by making people feel useful in pursuing a common goal within the farm. For example, people will be asked to take care of plants, collect vegetable and fruits for lunch, visit to farm animals, prepare labels for farm-stored food, and so on. Some activities will be repeated over time, albeit at an increasingly difficult level, in order to offer the subject, the possibility of learning them and carrying them out independently. Another key element drawn from the Montessori approach will be the continuous and attentive observation by the facilitator which allows the definition of an individualized plan of activities that are adequately stimulating and significant for the person (23). Based on observational data, the facilitator can adapt the difficulty of the tasks, encourage commitment, foster conditions for success and modify the proposed activity if the ongoing one does not align with the individual’s characteristics and preferences.

For the first time, the principles of the Montessori program will be adapted from the residential care context to an agricultural setting where individuals are in direct contact with nature, animals and the daily farm routines. This work of extrapolation and re-adaptation, as well as any element related to the application of Montessori principles to the project was curated by the Montessori International Centre of Perugia.

#### The training course

Before starting the project, the farm owners and facilitators will participate in a series of training meetings aimed at equipping them with the necessary skills to implement the project. In fact, according to the literature, it is crucial to provide operators with targeted and well-structured training to ensure that the principles of the Montessori educational philosophy are properly applied and lead to the intended benefits (24). The training course will be designed to develop competencies in the Montessori approach, social gerontology, cognitive disorders in the older adult, eco-social agriculture, and social farming planning. The course will consist of eight training sessions and will be led by psychologists, experts in social agriculture and a Montessori methodology expert.

#### Supervision meetings

To support and guide the farms and facilitators in managing the activities, monthly online and/or in-person supervision meetings will be held with INRCA psychologists and Montessori Centre experts. During these meetings, any challenges faced by the facilitators will be addressed, the proposals put forward by the participants and the necessary adjustments to the individualized plans based on satisfaction levels and involvement in the activities.

A checklist will be used to monitor the consistency and adherence of the proposed activities to Montessori principles. The checklist will evaluate, for instance, the inclusion of activities both on and off the farm, the procedures for welcoming participants upon arrival, the methods used to engage the five senses, the availability of rest areas, the presence of aromatic plants, animals, and trees among the proposed activities, the organization and upkeep of order within the farm’s indoor environment, and the strategies used to foster relationships and value each individual participant.

In addition, a WhatsApp group has been created among all the project partners to facilitate a rapid exchange of information, support or discussion when needed. According to the literature, in fact, it is crucial to support those delivering Montessori-based activities to help them manage any frustrations stemming from participants’ lack of engagement and necessary adjustments to the activities (24).

#### Team meeting

To establish a strong collaboration among the different partners involved in the project, periodic team meetings are planned. The primary goal of the project is to network diverse partners with varying interests: the University focuses on training younger generations on the topic of social agriculture, IRCCS-INRCA conducts scientific research to promote the quality of life and health of the older adult population, Coldiretti focuses on agricultural farms from an economic and social perspective, the International Montessori Centre deals with research on and applying the Montessori educational philosophy, farms that deal with hospitality, as well as the production and administration of local products. Additionally, throughout the project, the network of partners will gradually expand to form collaborations with other local organizations, such as ensuring transportation to and from the farm for the older adult participants involved. All these partners unite around the shared goal of promoting an innovative project to care for people with mild cognitive impairment, each contributing their own expertise. However, this alliance must be continuously refreshed and strengthened through the exchange of doubts, proposals, and coping strategies. These team meetings also address the co-creation of activities to be proposed within the project. Additionally, at the end of each prototype implementation cycle (Figure 1), the team will review any issues encountered during the 3 months of activity, the objectives achieved, the modification proposals from the participants, and will develop corrective interventions on the prototype deemed necessary for the next implementation cycle. Team meetings are scheduled at the beginning and end of each quarterly cycle. These meetings may be held more frequently based on specific needs.

### Intervention description

#### Frequency

Users will carry out the activities 3 days a week for 5 h a day (from 10:00 to 15:00) for a continuous quarterly cycle, repeatable for up to 9 months in total.

#### Activities

An individualized activity plan will be defined for each subject based on their individual psychological and cognitive characteristics, preferences, life history and current needs. The goal is to enhance the resources of each subject in order to promote their well-being and coping strategies. The activities will be co-constructed: IRCCS-INRCA will oversee the cognitive stimulation component, the International Montessori Centre will oversee the application of the Montessori principles through a humanistic approach and the farms will manage the social farming component. Meaningful activities drawn from the daily farm environment will be offered, including sensory and cognitive stimulation, as well as group socialization. Cognitive stimulation will be ecological-based not based on artificial tasks but on typical daily life activities (20).

#### Organization of the day on the farm

After an initial welcoming phase in a dedicated “corner” according to the Montessori principles relating to the preparation of educational spaces, the day on the farm will be dedicated to the activities outlined in the participants’ individualized plans. Sharing lunchtime will become an opportunity to socialize with the farmer’s family and create important bonds that will allow older people to feel part of an equal and cohesive group. At the end of the day the staff will say goodbye and remind participants of the next scheduled meeting.

To reach the farms and then return to their homes, participants will be able to use a transport service arranged in collaboration with local service providers.

#### Staff dedicated to reception

The target user with Mild Cognitive Impairment is characterized by the absence of significant impact of the diagnosed condition on autonomy in daily life (26, 27). This makes it possible for participants to carry out daily activities in a condition of autonomy. The activities are organized and conducted by the owner of the farm and by a support figure, called facilitator, possessing skills in social farming and hospitality. Facilitators will be selected and trained prior to the start of the project. The facilitator participates in the management of the activities and completes the individual daily record with significant data. The facilitator works in close contact with the farm owner and with psychologists, gerontology experts, and specialists in the Montessori approach. Instead, INRCA experts and those from Montessori International Centre will be present on the farm only on some days for the initial and final evaluation of the subjects involved in the project, to monitor the progress of the activities and to carry out supervision meetings.

## Measures and outcomes

The primary outcome will be the assessment of the positive and negative affective states. As previously highlighted, increasing positive affect and reducing negative affect in older adults with MCI appears to be associated with better health and improved social relationships, both of which are important for enhancing cognitive reserve and mitigating the effects of neuropathology.

Secondary outcomes will be changes in the perception of physical and mental health as indicators of perceived quality of life and the maintenance of cognitive level in the project participants. Finally, to evaluate the feasibility of the project, participants’ and their caregivers’ satisfaction will be assessed with the project activities and a qualitative and quantitative evaluation of the quality of engagement in the project activities will be conducted including the number of subjects who join and those who withdraw from the project. Primary and secondary outcomes as well as clinical assessment are described in Table 2.

**Table 2:** Outcomes and clinical assessment.

Outcome	Clinical assessment
Primary outcome	
Affective states	Positive and Negative Affect Schedule (PANAS)
Secondary outcome	
Quality of life	Short Form health survey (SF12)
Cognitive status	Montreal Cognitive Assessment (MoCa)
Participants satisfaction	Two ad hoc structured questionnaires
Appreciation by family members	Ad hoc structured questionnaire
Quality of engagement in activities	Ad hoc sheet on “Treatment monitoring”

### Data collection

Subjects will be contacted to schedule a visit with the clinical team for screening evaluation. During the screening session, each subject will undergo the Mini Mental State Examination (39), Tinetti’s Scale (40), Barthel Index (41) and the IADL scale (42). Once the compliance with the study’s inclusion and exclusion criteria are verified and the informed consent is obtained, the team will proceed with the baseline assessment. As this is a pilot study, no sample size calculation is planned; pilot studies typically involve a limited number of participants — in this case, 12.

At baseline, demographic data will be collected, the Positive and Negative Affect Schedule (PANAS) and the SF12 questionnaire will be administered to evaluate participants’ quality of life. Furthermore, participants will undergo the Montreal Cognitive Assessment (MoCa) to assess cognitive status.

At follow-up, participants’ evaluation will be assessed by means of Positive and Negative Affect Schedule (PANAS), the SF12 questionnaire and the Montreal Cognitive Assessment (MoCa). Satisfaction with the project will be evaluated through two structured questionnaires administered to both participants and their caregivers.

The assessment will be carried out at the beginning and at the end of each quarter (see Figure 1).

The scales which will be used during the evaluations are described below.

### Mini-mental state examination

The Mini-Mental State Examination (MMSE) is a brief screening tool developed as a clinical method to assess and grade cognitive impairment. The total score ranges from 0 to 30. Scores ≥24 are considered indicative of the absence of Major Neurocognitive Disorder (MND), scores between 18 and 23 suggest a mild stage of MND, scores between 11 and 17 indicate a moderate stage, and scores ≤10 are associated with severe MND. The raw score is adjusted based on the subject’s age and educational level (39).

### Tinetti’s scale or performance-oriented mobility assessment

Tinetti scale is a tool used to evaluate balance and gait performance. The test is clinically used to determine the mobility status of a subject or to assess changes in balance and gait time. The total POMA (POMA-T) consists of two sub-scales: the balance evaluation scale (“balance scale” or POMA-B) and the gait evaluation scale (“gait scale” or POMA-G). Scores equal to or <1 indicate a non-ambulatory subject, scores between 2 and 19 indicate a walking subject at risk of falling, scores equal to or greater than 20 indicate a walker with a low risk of falling (40).

### Barthel index

The Barthel Index is a questionnaire for systematically assessing Activity of Daily Living which asks about 10 basic ADLs (for example, personal hygiene, food intake, and toilet use). The maximum score of 100 indicates most independence, with a score of 0 indicating complete dependence in performing basic ADLs (41, 43).

### Instrumental activities of daily living scale (IADL)

The Instrumental Activities of Daily Living (IADL) scale assesses functional abilities in older adult individuals that are considered essential for maintaining independence. These activities include using the telephone, shopping, preparing meals, housekeeping, doing laundry, using transportation, taking medications, and managing finances. Each activity is scored from 0, indicating dependence, to 1, indicating independence. The total score ranges from 0 to 8 for women and from 0 to 5 for men, as three of the listed activities are traditionally not considered applicable for male respondents (41).

### Positive and negative affect schedule (PANAS)

The Positive and Negative Affect Schedule (44) is a measure of self-reported affect which assess the two most general emotional dimensions that describe affective experience and subjective well-being. The PANAS consists of two scales which are composed of 10 items each: the Positive Affect scale (PA) reflects the level of pleasant engagement (e.g., feeling enthusiastic, excited, active and determined) and the Negative Affect scale (NA) reflects a general dimension of individual distress (e.g., feeling scared, nervous, guilty and ashamed). The PANAS scales show excellent psychometric properties.

### Short form health survey (SF12)

The 12-item Short Form health survey, SF-12 (45–47), is a measure of health-related quality of life. It is an instrument with different weights for scoring physical and mental health, and measures health-related quality of life with 12 items categorized in eight areas: physical functioning, role physical, bodily pain, general health, vitality, social functioning, role emotional, and mental health. The raw scores of each item are coded, weighted, and summed into two scales: physical component summary score (PCS) and mental component summary score (MCS). Algorithms to calculate the value of the PCS and MCS indices of the SF-12 are available (47). Higher scores indicate better quality of life. The instrument will be used as in interviews.

### Montreal cognitive assessment (MoCA)

The Montreal Cognitive Assessment is a rapid short screening test (5–10′) widely used to assess mild cognitive impairment (48). It assesses multiple cognitive domains: executive functioning, attention, language, memory, visuospatial skills, and orientation. In Italy, the MoCA has been adapted and standardized (49, 50). It offers the possibility of calculating both a total score and subscores for each domain. Standardized norms are available for both the total score and the domain-specific subscores.

### Participants satisfaction ad hoc questionnaires

1. The first questionnaire consists of two items evaluated using a three-point Likert scale represented by a series of three facial expressions, ranging from a sad face (score = 1, “not very satisfied”) to a happy face (score = 3, “very satisfied”). The first item assesses the participants’ level of appreciation for the activities carried out during their stay on the farm. The second item evaluates whether the participants perceived a positive social and relational environment within the farm, and whether this made them feel welcomed and at ease. This questionnaire provides an important subjective measure of participant satisfaction and will be administered at the end of each three-month cycle of activity.
2. The second questionnaire is designed to assess the participant’s overall satisfaction either at the conclusion of the project or at the time they decide not to continue participating in the subsequent cycle of activities. It consists of six items. The first three are evaluated using a Likert scale ranging from 0 to 10, where 0 represents the lowest score, 10 the highest, and 5 a neutral score. These items investigate: (1) how the participant evaluates the overall experience and the reasons behind their rating; (2) whether they perceive any changes in their physical condition; and (3) whether they notice any changes in their emotional or mental well-being since participating in the project. The remaining three items are open-ended questions designed to gather more in-depth feedback. These questions explore the participant’s general comments, the reasons that led them to take part in the project, and any suggestions for improving its organization (e.g., timing, activities, or other aspects).

### Caregivers satisfaction ad hoc questionnaire

The questionnaire is designed to assess the satisfaction of the informal caregiver at the end of the entire project or in any case when the subject decides not to participate in the activities in the following quarter. The questionnaire is composed of six items. Three of them are assesses with a Likert scale from 0 to 10 where 5 is the neutral score, 0 is the worst score and 10 is the best score. The items concern: how the caregiver evaluates the experience of his/her loved one in the project and why; if they observe any change in the physical conditions and then in the emotional or mental conditions of their loved one compared to before starting to participate in the project activities. The other three items are open questions on more general comments, on what prompted the caregiver to have their loved one participate in the project and if they would change something in the organization of the project itself (time, activities, other).

### Ad hoc sheet on “treatment monitoring”

In order to evaluate and, if necessary, redefine the individualized activity plan developed for each patient, a dedicated has been created to enable continuous quantitative and qualitative assessment. The *ad hoc* sheet “Treatment monitoring” will be filled out by the facilitator based on constant observation of the participant during the activities in the farm.

The quantitative section of the observation sheet evaluates the following parameters: interest, communication, enjoyment, and mood. Each of these factors is assessed using a 5-point Likert scale, where 1 represents the lowest level (e.g., “no interest,” “did not communicate during the meeting,” “did not show enjoyment,” “very low mood”) and 5 indicates the highest level (e.g., “showed a lot of interest,” “communicated very well during the meeting,” “had a lot of fun during today’s session,” “very good mood, appeared relaxed and happy”). This scale allows for a nuanced understanding of the participant’s engagement and emotional state throughout the activities.

Furthermore, the facilitator will have the opportunity to qualitatively note in it the significant elements of participation in the individualized treatment with a description of the events and emotional behaviors of the user like as level of participation, concentration, boredom, and behaviors of distancing from the activity. The form concludes by asking the facilitator to identify the Montessori markers that define the daily activities conducted with each individual. Examples of Montessori markers include, for instance, the assessment of the participant’s emotional state upon arrival at the farm, opportunities to exercise free choice during activities, the repetition of proposed tasks to support learning and independent execution, responses to external distracting stimuli, and the facilitator’s need to implement strategies to support concentration.

### Follow-up evaluation

The follow-up survey will take place at the end of the 3-month intervention and constitutes the final phase of the first cycle of prototype implementation. All measures from the initial screening will be repeated to check for any changes in the participants’ condition. In particular, it will be noted whether the subjective well-being has increased since the first survey, so whether the positive affects has increased and negative affects has decreased and if quality of life as increased. Moreover, it will be noted whether the participants and their caregivers are satisfied with the project through two *ad hoc* satisfaction questionnaires. This evaluation will be useful in order to identify any changes likely to improve the intervention.

Finally, after the three implementation cycles, the number of subjects who participated in one or more project cycles and the number of subjects who abandoned the project and the reasons for such dropouts will also be evaluated.

### Statistical analysis

Data will be expressed by mean and standard deviation for continuous variables and by frequency and percentages for categorical ones.

Comparisons of all the scale variables of interest in the time (baseline vs. 3 months follow-up) will be performed using paired samples t test. We could also categorize some scales using specific cut-off and could evaluate if there has been a change of category over time by chi-square test for example.

Statistical significance will be set at *p* < 0.05.

All analyses will be performed using IBM SPSS Statistic for Windows, Version 24.0 (IBM Corp., Armonk, NY, USA).

## Discussion

The aim of this study is to evaluate an individualized social intervention for individuals with mild cognitive impairment (MCI) in a farm-based setting. Research indicates that individuals with MCI can significantly benefit from proactive interventions that enhance the quality of their aging process, potentially reducing the risk of developing dementia while preserving their autonomy for as long as possible (10). Furthermore, literature suggests that promoting healthy aging in people with MCI requires more than just supporting their subjective well-being. It is essential to create an environment conducive to preventing or delaying the progression to dementia (10). Maintaining an active lifestyle—both cognitively and socially—is a crucial component of this approach, and the environment in which individuals with MCI engage should be structured to facilitate this goal (10, 28). In this context, the farms involved in the project provide a stimulating yet relaxing and non-medicalised environment, where participants can engage in a variety of therapeutic activities. Research suggests that the natural setting of the farm offers participants enjoyable and purposeful tasks, while also promoting greater autonomy and the freedom to make choices about their actions (16, 28). Additionally, taking part in social farming activities helps individuals develop a sense of accomplishment and satisfaction through the tangible nature of the tasks, as well as fostering a sense of belonging to a community where meaningful social connections can thrive (29). Consequently, in order to propose activities suitable for everyone and promote trust between peers, small homogeneous groups of individuals with MCI will be formed through the assessment of cognitive decline during the initial screening. Furthermore, the farms involved in the project will aim to promote people’s emotional well-being and quality of life through continuous engagement in daily life-related and meaningful activities by taking advantage of direct contact with nature, its slow rhythms and repetitive routines (28). The connection between the components of social agriculture and the Montessori principles will allow the project to promote activities well calibrated on the cognitive and psychosocial characteristics of each participant through careful observation. A recent review emphasizes how horticultural therapy, the Montessori methods for dementia, sensory and reminiscence therapies have many overlapping aspects and together they appear to be useful in decreasing agitation and improving the physical, cognitive, emotional and social functions of people with dementia (33). The configuration of an innovative service, both in terms of the approach to individuals with mild cognitive impairment and as a relief service for their family members, positions the farm as a key player in a new model of rural welfare. This model offers positive economic benefits for both the farms and public administrations through the optimisation of resources. Consequently, the Silver Agri Age project aims to establish an innovative pilot experience of social care for people with MCI, which can later serve as a best practice model to be extended to other farms looking to implement similar initiatives. While the sample size of 12 participants is relatively small, it is important to note that this is a pilot study, and as such, it is designed to serve as a foundation for future, larger-scale studies with an appropriately sized sample.

### Potential risks, burdens, and benefits for participants

We expect that the individualized program aimed at older adult people with mild cognitive impairment will improve the emotional well-being and quality of life of participants. With regard to potential risks, the target audience with Mild Cognitive Impairment is characterized by the absence of significant impact of the diagnosed condition on normal daily life autonomy and therefore does not present additional risks compared to a normal older adult population. As a precaution, however, participants are always advised to consult with their GP to discuss any doubts with him, and to inform him when he recognizes a sudden worsening of symptoms. Finally, the clinical nature of MCI may present challenges for farms and facilitators, such as cognitive impairments (e.g., forgetting appointments) and potential psychological or behavioral issues (e.g., irritability, aggression, or melancholy). These factors necessitate the provision of personalized and ongoing support by the farms, posing a considerable burden given that it must be delivered in a non-healthcare environment.

It should be noted that pursuant to art. 12 of the Marche regional law of 14 November 2011 n. 21 containing the “Regional provisions on the multifunctionality of the farm and diversification in agriculture,” the Marche regional organizational structure has established the regional list of agritourism operators (EROAS) who performed the activities referred to in art. 9 of the same law (educational farm). The operators who will take part in the project will be registered in the above list and will comply with all the rules that fall within the scope of the specific activities.

### Expected impact

#### Older adult participants

We anticipate that the individualized program will enhance the emotional well-being and quality of life for participants with MCI. By engaging in activities on the farm and sharing time with the diverse individuals who frequent it, participants will feel less isolated and more integrated into a group that genuinely values their experiences and opinions. Furthermore, we expect that participation in meaningful, purpose-driven activities, relevant to maintaining autonomy in daily life and adequately stimulating can keep their cognitive state stable and offer them the opportunity to redefine a purpose that animates their days. Studies show that having a purpose in life allows you to maintain your well-being and improve your quality of life (51).

#### Secondary end-users (informal caregivers)

We expect that family caregivers can benefit from the participation of their older adult loved ones in the project, first of all because they can have more time off from care because of their family member is safe and cared for on the farm. Furthermore, caregivers could have a lower care burden thanks to the fact that their loved ones are more serene, satisfied in their needs and therefore less demanding.

### Limitations

A limitation of this study is its small sample size, although this is common in pilot studies. Nevertheless, this feasibility study will offer valuable organizational insights to support the generalizability and scalability of the service. For the project funders, it is crucial to understand the prerequisites for expanding this social agriculture initiative to as many farms as possible within the Marche region. Based on user feedback regarding satisfaction and well-being, necessary adjustments to both the farms and the project structure will be evaluated to accommodate a larger number of participants.

A further methodological limitation of this study is the omission of a control group from the research protocol. The limited demand for diagnostic evaluation among individuals with mild cognitive impairment (MCI) until significant impairment in daily living activities occurs restricts the pool of eligible participants in this feasibility assessment phase. As a result, the incorporation of a control group will be deferred to subsequent studies, which will leverage collaborations with local healthcare facilities to implement a more extensive recruitment strategy, ultimately enhancing the methodological rigor and validity of the investigation.
